# Endothelial Glycocalyx Anomalies and Ocular Manifestations in Patients with Post-Acute COVID-19

**DOI:** 10.3390/jcm13237272

**Published:** 2024-11-29

**Authors:** Georges Azar, Youssef Abdelmassih, Sophie Bonnin, Damien Guindolet, Vivien Vasseur, Francine Behar Cohen, Dominique Salmon, Martine Mauget-Faÿsse

**Affiliations:** 1 Rothschild Foundation Hospital, 75019 Paris, France; 2 Ophthalmology Department, Cochin Hospital, 75014 Paris, France; 3 Infectious and Tropical Diseases Department, Hotel Dieu, 75004 Paris, France

**Keywords:** adaptive optics, GlycoCheck, long COVID, post-acute COVID-19, retinal ischemia

## Abstract

**Objectives**: To report ophthalmological and microvascular findings in patients with post-acute COVID-19. **Methods**: In this prospective, monocentric cohort study, we included patients with post-acute COVID-19 who presented with ophthalmological symptoms. All patients underwent indocyanine green angiography (ICGA), OCT, OCT-angiography, adaptive optics, and GlycoCheck assessments. **Results:** We included 44 patients, predominantly female (81.8%), with a mean age of 47.5 ± 11.5 years. Key ICGA findings revealed hyperreflective dots in 32 eyes (36.4%) and hemangioma-like lesions in 7 eyes (8.0%). Capillary non-perfusion in the superficial capillary plexus (SCP) and deep capillary plexus (DCP) was observed in 42 eyes (47.7%) and 21 eyes (23.9%), respectively. Eyes with hyperreflective dots exhibited a lower perfused boundary region (PBR), while those with superficial punctate keratitis showed a higher PBR (*p* = 0.02 and *p* = 0.002, respectively). Eyes with capillary non-perfusion in the SCP displayed lower capillary densities (CD4, CD5, and CD4-6; *p* = 0.001, 0.03, and 0.03, respectively), and eyes with non-perfusion in the DCP had lower CD4 (*p* = 0.03). A negative correlation was identified between capillary density and the wall-to-lumen ratio. **Conclusions:** Patients with post-acute COVID-19 demonstrate both retinal and choroidal vascular anomalies. Ocular pathology was associated with reduced capillary density. These injuries appear to stem more from microvascular disruptions than from persistent glycocalyx abnormalities.

## 1. Introduction

Soon after the onset of the coronavirus disease 2019 (COVID-19) pandemic in Wuhan, China, in 2019, the term “post-acute COVID-19” (or “long COVID syndrome”) began gaining recognition within the scientific and medical communities [1,2]. The National Institute for Health and Care Excellence (NICE) defines this condition as signs and symptoms that develop during or following a COVID-19 infection, persist for more than three months after onset, and are not explained by alternative diagnoses [3]. The persistent and diverse clinical symptoms of post-acute COVID-19 pose a growing challenge for healthcare systems globally, representing a significant health concern. Patients with this condition experience multi-organ complications and enduring symptoms, including cardiorespiratory (e.g., chronic cough, shortness of breath, chest tightness), sensory (e.g., loss of taste and smell), and neurological (e.g., cognitive dysfunction and extreme fatigue) issues [4,5].

Similarly, Severe Acute Respiratory Syndrome-Coronavirus-2 (SARS-CoV-2)-related ocular findings have been recognized, with conjunctivitis being the most commonly reported [6]. Additionally, posterior segment manifestations may arise, either through direct viral entry or as an indirect inflammatory response. These manifestations include cotton wool spots (CWS), intraretinal hemorrhages, dilated and tortuous vessels, retinal vein occlusions, acute macular neuroretinopathy (AMN), paracentral acute middle maculopathy (PAMM), and COVID-19-associated choroidopathy [7,8,9,10]. Although ocular manifestations of SARS-CoV-2 infection have been extensively documented, the pathophysiology, prevalence, and clinical course of these findings in post-acute COVID-19 syndrome remain poorly understood, and global studies are still limited [11].

In addition, several studies have reported endothelial glycocalyx (EGL) injury associated with severe COVID-19 infection [12]. The EGL plays an essential role in regulating vascular permeability and cell adhesion, providing anti-inflammatory and antithrombotic functions, and acting as a mechanosensor for hemodynamic shear stress [13]. While measuring the EGL and assessing microvasculature size has been challenging, a new non-invasive technique has been developed using the GlycoCheck system. This system involves a handheld camera (Capiscope handheld, KK Research Technology Ltd.) positioned under the tongue to capture in vivo video recordings of capillary blood flow, which is then analyzed with GlycoCheck software [14].

Little is known about EGL and microvascular injury in post-acute COVID-19, its implications in ocular manifestations, and the underlying putative pathophysiological mechanisms related to this injury.

The aim of this study was to report on ocular manifestations of post-acute COVID-19, to evaluate the EGL and microvascular damage using the GlycoCheck system, to correlate these damages with the ocular manifestations, and finally elaborate potential pathophysiological mechanisms.

## 2. Materials and Methods

### 2.1. Study Design and Participants

In this observational prospective monocentric cohort study we included patients with post-acute COVID-19 and ocular symptoms, followed at the infectious and tropical diseases department, Hotel Dieu, Paris and referred to the Rothschild Foundation Hospital, Paris for a thorough ophthalmological examination and GlycoCheck test, between 7 February 2022 and 30 June 2022. Patients included in this study met the following criteria: adults (age > 18 years); confirmed diagnosis of COVID-19 via SARS-CoV-2 reverse transcriptase-polymerase chain reaction (RT-PCR) of a nasopharyngeal swab and/or SARS-CoV-2 antibody testing; presence of clinical manifestations consistent with post-acute COVID-19 as defined by the NICE guidelines [3]; and reported ophthalmological symptoms. The study received approval from the institutional review board and ethical committee (N° IDRCB: 2021-A02604-37) and was conducted in accordance with the Declaration of Helsinki. All participants signed a written informed consent form outlining the study’s objectives and the procedures they would undergo.

### 2.2. Data Collection

Patient demographic characteristics and hospitalization details were documented, including age, sex, systemic comorbidities (e.g., hypertension, diabetes mellitus, heart disease, autoimmune disease, and thromboembolic disease), and medications taken (such as tocilizumab, steroids, and diuretics). At presentation, all patients received a comprehensive ophthalmologic evaluation, including best-corrected visual acuity (BCVA) and a detailed slit-lamp examination (SLE). The SLE recorded tear film break-up time (abnormal if exceeding 5 s between the last blink and the first signs of lipid layer disruption on the corneal surface), presence of superficial punctate keratitis (SPK), and blepharitis. Intraocular pressure (IOP) was measured, and fundus ophthalmoscopy was performed. Additional imaging and diagnostic tests included ultra-wide field imaging (Optos PLC, Dunfermline, UK), infrared scanning laser ophthalmoscopy (SLO), spectral domain-optical coherence tomography (SD-OCT) B-scan, OCT-angiography (OCT-A), and indocyanine green angiography (ICGA) (Heidelberg Engineering, V1.10.12.0, 69115 Heidelberg, Germany). Adaptive optics (AO) SLO was conducted using the rtx1TM device (Imagine Eyes, Orsay, France). Lastly, all participants underwent sublingual video microscopy with a sidestream dark field (SDF) camera (CapiScope HVCS, KK Technology, Honiton, UK) integrated with GlycoCheck 5.2 software (Microvascular Health Solutions Inc., Alpine, UT, USA) for microvascular assessment.

### 2.3. Optical Coherence Tomography (B-Scan) and OCT-A

The OCT examination included macular line scans and volume scans. Additional line scans were performed during ICGA when anomalies were detected. Subfoveal choroidal thickness was manually measured using enhanced depth imaging (EDI) scans. The presence of pachyvessels was also assessed [15]. OCT-A volumes were acquired over a 9 × 9 mm field of view. The superficial and deep capillary plexuses (SCP and DCP) were automatically segmented. Capillary non-perfusion areas in both the SCP and DCP were assessed manually.

### 2.4. Indocyanine Green Angiography (ICGA)

ICGA images of the posterior pole and peripheral regions were obtained during the early, intermediate, and late phases. Two retinal specialists (SB and MMF) independently analyzed the images. The presence of the following features was assessed: hyperreflective dots, choroidal hemangioma-like lesions, and choroidal vessel leakage [10].

### 2.5. rtx1 Examination Protocol and Parameter Evaluation

For image acquisition, the reference site was a segment of the supero-temporal artery in the right eye, at least 250 µm in length, with an inner diameter of at least 50 µm, free of bifurcations, and located one disc diameter from the optic disc. The highest-quality images captured were selected and analyzed using the AOdetectArtery image processing and recognition 3.0 software to assess the retinal vasculature [16]. Measurements of total vessel diameter, wall thickness (WT), and lumen diameter were recorded. The wall-to-lumen ratio (WLR) and wall cross-sectional area (WCSA) were automatically calculated.

### 2.6. In Vivo Assessment of the Microcirculation and Glycocalyx Dimensions

An SDF camera coupled with the GlycoCheck software, which assesses the endothelial glycocalyx layer (EGL), was used to visualize the sublingual microvasculature and record the movement of red blood cells within the microvessels, as previously described by Rovas et al. [17]. The following parameters were calculated: perfused boundary region (PBR, in μm), capillary density (CD, in 10^−2^ mm/mm^2^), absolute, static, and dynamic capillary blood volume (CBV_absolute_, CBV_static,_ and CBV_dynamic_); and, finally, microvascular health score (MVHS, in points) [4].

### 2.7. Statistical Analysis

Continuous variables are presented as means with standard deviations, while qualitative variables are presented as frequencies and percentages. For continuous variables, statistical analysis was performed using either an unpaired t-test or the Wilcoxon–Mann–Whitney test, depending on the data distribution. For qualitative variables, statistical analysis was performed using the two-tailed Pearson’s χ^2^ test (with or without Yates’ correction) or Fisher’s exact test. The Spearman rank correlation coefficient was used to assess correlations between ocular findings, adaptive optics results, and sublingual microcirculation findings obtained with the GlycoCheck. All statistical tests were two-sided, with significance considered for *p*-values < 0.05. Analyses were conducted using SPSS version 26 (IBM Corporation, Armonk, NY, USA), and figures were prepared using GraphPad Prism version 8.4.3 (GraphPad Software Inc., San Diego, CA, USA).

## 3. Results

### 3.1. Study Population and Characteristics of Ocular Findings

A total of 44 patients (88 eyes) [36 women (81.8%) and 8 men (18.2%), *p* < 0.05] with a mean age at presentation of 47.5 ± 11.5 years was included. The patients were examined on average 23.0 ± 3.8 months after the initial infection. Table 1 reports on the baseline characteristics, and medical records of included patients. Twelve patients (27.3%) received oral or intravenous steroid therapy within the three months preceding presentation and eleven (25.0%) had a history of autoimmune disease.

With regard to ophthalmological findings, anterior and posterior segment manifestations are listed in Table 2. The mean central choroidal thickness was 352.2 ± 110.9 μm and 52 eyes (59.1%) had pachyvessels detected on SD-OCT. Capillary non-perfusion within the SCP was found in 42 eyes (47.7%) and within the DCP in 21 eyes (23.9%). The average mean vascular density was 0.068 ± 0.007 and 0.057 ± 0.010 in the SCP and DCP, respectively. The average of sum vascular density was 6.5 ± 1.1 and 2.9 ± 0.68 in the SCP and DCP, respectively.

**Figure 1:**
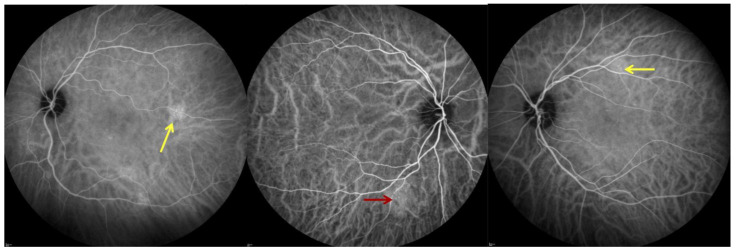
Indocyanine green angiography (ICGA) showing hyperreflective dots defined as pinpoint leakage visible in the intermediate- or late-phases mostly seen in the retinal mid periphery (yellow arrows); choroidal hemangioma-like lesion, defined as a well-circumscribed hyperfluorescent choroidal area with pinpoints in the ICGA mid-phase (red arrow).

With regard to ICGA signs, hyperpermeability was detected in 47 eyes (53.4%), hyperreflective dots in 32 eyes (36.4%), and hemangioma-like lesions in 7 eyes (8.0%). Figure 1 shows ICGA anomalies.

### 3.2. Adaptive Optics

**Figure 2:**
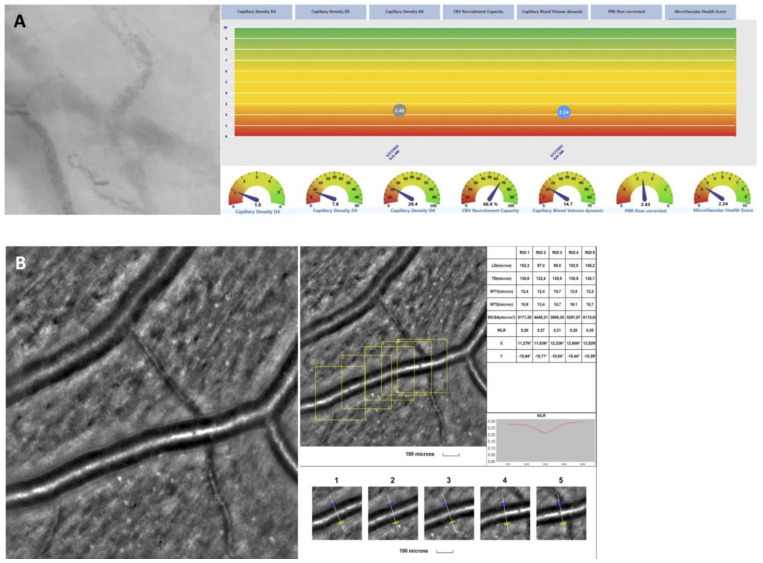
(**A**): In vivo assessment of the sublingual microcirculation and glycocalyx dimensions using the GlycoCheck 5.2 software and assessment of retinal vessel microstructure. (**B**): adaptive optics showing a representative image from the retinal artery analysis with an example of microvascular changes that could be seen in post-acute COVID-19 patients with ocular manifestations.

Overall, the mean WLR was 0.27 ± 0.05 μm, the mean WCSA was 3931.4 ± 907.7 μm^2^, and the mean WT was 12.1 ± 1.9 μm. Table 3 reports on AO parameters. A representative image from the retinal artery analysis showing an example of microvascular changes with increased WLR and thickening of arteriole walls is shown in Figure 2.

### 3.3. Sublingual Glycocalyx Parameters

The mean PBR was 2.2 ± 0.2 μm, the mean CD4-6 was [23.9 ± 12.2] ×10^−2^ mm/mm^2^. When further divided according to vessel diameter for a μm-precise analysis [i.e., 4 μm (D4), 5 μm (D5), and 6 μm (D6)], the mean capillary density in each vessel diameter group was [0.8 ± 0.7] ×10^−2^ mm/mm^2^, [6.2 ± 3.9] ×10^−2^ mm/mm^2^, and [17.9 ± 8.3] ×10^−2^ mm/mm^2^, respectively. The mean CBV_absolute_ was 6.1 ± 3.1 μm^3^, the mean CBV_static_ was 1.2 ± 0.2 μm^3^, and the mean CBV_dynamic_ 13.2 ± 8.4 μm^3^. The mean MVHS index was 2.4 ± 1.6. Table 3 reports on endothelial parameters measured with the GlycoCheck. Figure 2 shows a presentation of GlycoCheck result.

### 3.4. Correlation Analysis

The PBR was higher in eyes with SPK on SLE (2.3 vs. 2.2; *p* = 0.002) and lower in eyes with hyperreflective dots on ICGA (2.2 vs. 2.3; *p* = 0.02). Seventy-seven percent of eyes with hyperreflective dots were of patients with a history of autoimmune disease (*p* = 0.001). Eyes with capillary non-perfusion in the SCP had significantly lower CD4 (0.53 ± 0.4 vs. 0.98 ± 0.73; *p* = 0.001), CD5 (5.1 ± 2.5 vs. 6.8 ± 4.3; *p* = 0.03), and CD7 (20.7 ± 8.7 vs. 26.1 ± 13.2; *p* = 0.03). Eyes with capillary non-perfusion in the DCP had significantly lower CD4 (0.56 ± 0.4 vs. 0.84 ± 0.7; *p* = 0.03) but higher total density (196 ± 57.4 vs. 157 ± 60.9; *p* = 0.01).

A negative correlation was found between WLR and CD4 (pearson coefficient: −0.25; *p* = 0.02), CD5 (pearson coefficient:−0.26; *p* = 0.02), CD6 (pearson coefficient: −0.29; *p* = 0.009), and CD7 (pearson coefficient:−0.29; *p* = 0.009).

## 4. Discussion

The full spectrum of post-acute COVID-19 related signs and comorbidities, particularly patients’ ophthalmological manifestations, should be understood by clinicians, meaningfully facilitating the basic understanding of the disease. This study aims to describe the presence of ocular manifestations in patients with post-acute COVID-19 syndrome and investigate their potential correlations with sublingual glycocalyx parameters measured using the GlycoCheck. Based on these potential correlations, we also sought to explore the underlying pathophysiological mechanisms that may contribute to the disease’s natural history.

To date, the mechanisms of post-acute COVID-19 remain poorly understood. It has been reported that SARS-CoV-2 enters host cells through the entry receptor, angiotensin-converting enzyme (ACE)-2. The mechanisms associated with SARS-CoV-2 infection include: direct virus-mediated cell damage; dysregulation of the renin-angiotensin-aldosterone system (RAAS) following the downregulation of ACE-2 receptors; damage to the EGL and endothelial cells, leading to thromboinflammation; dysregulation of the immune response; and alterations in the autonomic nervous system [9,10,18].

Dry eyes and keratitis were among the most frequent ocular manifestations found in patients. Among the parameters assessed with the GlycoCheck, PBR strongly correlated with the presence of SPK (*p* = 0.002). Ocular surface diseases such as dry eyes, meibomian gland dysfunction, and tearing are frequently reported both during acute and post-acute COVID-19. In a metanalysis of ocular surface manifestations that reviewed 16 studies and involved a total of 2347 patients with acute COVID-19, ocular pain was found 31.2% of patients, discharge in 19.2%, and redness in 10.8% [19]. Similarly, ocular surface disturbance was also frequent in the post COVID-19 period, seemingly associated with higher viral load and the oxygen requirement [20]. In our cohort, patients with SPK had a higher PBR, and therefore a worse EGL than eyes without. The higher PBR could be secondary to a more severe COVID-19 infection that could affect the ocular surface, meibomian gland, and the autonomous nervous system.

In our population of patients with ophthalmological symptoms and post-acute COVID-19, mean PBR was 2.2 ± 0.2 μm, which was within the normal range. Several studies have reported on a partial recovery of glycocalyx function in the months following the COVID-19 infection [21,22]. Osiaevi et al., who evaluated the microvasculature of post-acute COVID-19, reported a complete restoration of glycocalyx function shown by the normalization of PBR about 1.5 years following the initial infection [23]. This was not corroborated by Ikonomidis et al. who, despite finding a partial improvement of myocardial work and vascular dysfunction, showed a persistent glycocalyx dysfunction and PBR increase 1 year after initial infection [24]. We can assume that acute COVID-19 infection strongly affects the EGL, which progressively improves with time and regains normal function; however, this can take several months or years.

EGL dysfunction during acute COVID-19 can lead to thromboembolic events. In fact, capillary non-perfusion in both the SCP and DCP was frequently observed, occurring in 47.7% and 23.9% of patients, respectively. Patients with capillary non-perfusion had significantly lower capillary density, particularly in smaller capillaries of 4 and 5 μm in diameter. Both acute and post-acute COVID-19 are multisystemic vascular diseases, where microvascular impairment appears to play a crucial role [25]. Osiaeavi et al. found that patients with post-acute COVID-19 had significantly lower vascular density compared to healthy controls, with μm-precise analysis showing that the decrease in vascular density primarily affected very small capillaries [23]. Their data strongly suggest that long COVID leads to persistent capillary rarefaction, even 18 months after infection [23]. Capillary rarefaction appears to be associated with post-acute COVID-19 and correlates with capillary non-perfusion in the retina. In our cohort, the mean microvascular health score was 2.4 ± 1.6, which is lower than that in the normal population. This index seems to be more strongly affected by capillary density than by perfused boundary region values.

A strong association was identified between PBR and hyperreflective dots, which appeared as drusen-like deposits on SD-OCT and were more commonly observed in patients with autoimmune diseases. These drusen-like deposits may be linked to dysregulation of the complement cascade, similar to mechanisms observed in AMD. In AMD, complement dysregulation and abnormal complement production, both locally and systemically, have been implicated in disease development and severity [26]. Notably, recent studies indicate that post-acute COVID-19 patients exhibit dysregulation in the terminal complement system, with ongoing activation of the classical and alternative complement pathways [27]. This chronic inflammatory response leads to endothelial cell damage, potentially resulting in drusen formation. In fact, it has been shown that endothelial cell damage in the choroid results in drusen formation and contributes to AMD pathogenesis [28].

Finally, we found a negative correlation between CD and WLR, but no correlation was found between the PBR and AO variable. The EGL attenuation may not be detected because of the resolution of the rtx1 machine. In fact, the in vivo dimension of the glycocalyx in healthy individuals varies from 500 nm to 2 μm while the lateral resolution of the rtx1 camera ranges from 850 nm to 1.6 μm [16,29]. However, eyes with low CD had a higher WLR. In fact, the WLR was found to be higher in patients with cardiovascular diseases and diabetes mellitus. Cífková et al. found WLR to be increased in newly detected individuals with hypertension and in untreated hypertensive patients which reflect early vascular damage [30]. Similarly, Zaleska-Żmijewska et al. reported an increase in WLR in patients with diabetic retinopathy [16].

This study has limitations within which our findings need to be carefully interpreted. First, it includes a relatively small sample of patients, and its monocentric design does not necessarily extrapolate the data to other units and the general population. Second, caution is needed when interpreting the GlycoCheck parameters due to the absence of a case-matched control group and well-established normative data. Consequently, we compared our findings to those reported in other populations in literature. The results of our study should be corroborated in further case-matched studies.

## 5. Conclusions

In conclusion, patients with post-acute COVID-19 have frequent ophthalmological anomalies mainly including ocular surface disease and retinal capillary non-perfusion. They seem to have normalization of the glycocalyx function but an abnormal CD and microvascular bed. The abnormal capillary density was associated with retinal capillary non-perfusion and with high WLR. The symptoms reported by post-acute COVID-19 patients seem to be the result of microvascular rarefaction and damage to the capillary bed. Longer follow-up and case control studies remain crucial to better understanding this disease and for evaluation of the reversibility of the microvascular anomalies found.

## Figures and Tables

**Table 1:** Demographic characteristics of included patients. SD: standard deviation.

	Overall Population (*n* = 44)
Age in years (Mean ± SD)	47.5 ± 11.5
**Sex:**	
**Female (%)**	36 (81.8%)
**Male (%)**	8 (18.2%)
**Interval following initial infection in months (Mean ± SD)**	>23.0 ± 3.8
**Diabetes mellitus (%)**	1 (2.3%)
**Hypertension (%)**	6 (13.6%)
**Cardiovascular disease (%)**	1 (2.3%)
**Thromboembolic disease (%)**	0 (0%)
**Allergy (%)**	18 (40.9%)
**Auto-immune disease (%)**	11 (25.0%)
**Treatment**	
**Corticosteroids (%)**	12 (27.3%)

**Table 2:** Ocular manifestations of included patients. OCT-A: optical coherence tomography angiography; SD: standard deviation; TBUT: tear break-up time.

	Eyes (*n* = 88)	Female (*n* = 72)	Male (*n* = 16)	*p*-Value
**TBUT < 5 s (%)**	39 (44.3%)	31 (43.1%)	8 (50.0%)	0.61
**Keratitis (%)**	30 (34.1%)	27 (37.5%)	3 (18.8%)	0.15
Blepharitis (%)	17 (19.3%)	15 (20.0%)	2 (12.5%)	0.44
Choroidal thickness **in μm (Mean ± SD)**	325.2 ± 110.9	324.8 ± 114.3	326.9 ± 97.8	0.94
Presence of pachyvessels (%)	52 (59.1%)	42 (58.3%)	10 (62.5%)	0.76
OCT-A abnormal superficial capillary plexus (%)	42 (47.7%)	33 (45.8%)	9 (56.3%)	0.45
OCT-A abnormal deep capillary plexus (%)	21 (23.9%)	17 (23.6%)	4 (25.0%)	0.91
Hyperreflective dots (%)	32 (36.4%)	26 (36.1%)	6 (37.5%)	0.92
Hemangioma-like lesions (%)	7 (8.0%)	6 (8.3%)	1 (6.3%)	0.78
Hyperpermeability (%)	47 (53.4%)	39 (54.2%)	8 (50.0%)	0.76

**Table 3:** Adaptive optics measurements and vascular endothelial glycocalyx (GlycoCheck) parameters of included patients. CBV_dynamic_: dynamic capillary blood volume; MVHS: microvascular health score; PBR: perfused boundary region; SD: standard deviation; WCSA: cross-sectional area of the vascular wall; WLR: wall-to-lumen ratio; WT: single arteriolar wall thickness.

Adaptive Optic (88 Eyes)	
**WLR, μm (mean ± SD)**	0.3 ± 0.0
**WCSA, μm^2^ (mean ± SD)**	3931.4 ± 907.7
**WT, μm (mean ± SD)**	12.1 ± 1.9
**GlycoCheck (44 patients)**	
**Number of exams per patient (mean ± SD)**	14.6 ± 8.7
**PBR, μm (mean ± SD)**	2.2 ± 0.2
**Flow total density D4–D25, ×10^−2^ mm/mm^2^ (mean ± SD)**	166.8 ± 63.0
**Capillary density D4–D6, ×10^−2^ mm/mm^2^ (mean ± SD)**	23.9 ± 12.2
**CBV_dynamic_ (mean ± SD)**	13.2 ± 8.4
**MVHS (mean ± SD)**	2.4 ± 1.6

## Data Availability

The original contributions presented in the study are included in the article, further inquiries can be directed to the corresponding author.
